# Distinct Cold Acclimation of Productivity Traits in *Arabidopsis thaliana* Ecotypes †

**DOI:** 10.3390/ijms23042129

**Published:** 2022-02-15

**Authors:** Barbara Demmig-Adams, Stephanie K. Polutchko, Christopher R. Baker, Jared J. Stewart, William W. Adams III

**Affiliations:** 1 Department of Ecology and Evolutionary Biology, University of Colorado, Boulder, CO 80309, USA; stephanie.polutchko@colorado.edu (S.K.P.); jared.stewart@colorado.edu (J.J.S.); william.adams@colorado.edu (W.W.A.III); 2 Department of Plant and Microbial Biology, Howard Hughes Medical Institute, University of California, Berkeley, CA 94720, USA; cbaker@berkeley.edu

**Keywords:** daylength, excitation pressure, freezing, growth, high light, Lhcb, nonphotochemical quenching, phloem, photosynthetic capacity, xylem

## Abstract

Improvement of crop climate resilience will require an understanding of whole-plant adaptation to specific local environments. This review places features of plant form and function related to photosynthetic productivity, as well as associated gene-expression patterns, into the context of the adaptation of *Arabidopsis thaliana* ecotypes to local environments with different climates in Sweden and Italy. The growth of plants under common cool conditions resulted in a proportionally greater emphasis on the maintenance of photosynthetic activity in the Swedish ecotype. This is compared to a greater emphasis on downregulation of light-harvesting antenna size and upregulation of a host of antioxidant enzymes in the Italian ecotype under these conditions. This differential response is discussed in the context of the climatic patterns of the ecotypes’ native habitats with substantial opportunity for photosynthetic productivity under mild temperatures in Italy but not in Sweden. The Swedish ecotype’s response is likened to pushing forward at full speed with productivity under low temperature versus the Italian ecotype’s response of staying safe from harm (maintaining redox homeostasis) while letting productivity decline when temperatures are transiently cold. It is concluded that either strategy can offer directions for the development of climate-resilient crops for specific locations of cultivation.

## 1. Introduction

### 1.1. Climate Resilience

The need to develop “climate-resilient crops for improving global food security and safety” is intensifying [1] as extreme weather events become more common, with greater heat in the summer, more frequent and lasting droughts that can extend through more than one season, as well as late-spring/early-fall cold spells [2]. Climate-resilient crops are needed that maintain high productivity despite less predictable weather patterns and under unique combinations of environmental stresses [3]. Even evasive agricultural strategies require the concomitant development of new crop lines. For example, early sowing of summer crops is used to shift crop production to cooler months of the year, thus evading the most intense summer heat/drought and improving water-use efficiency [4]. Another evasive strategy involves the relocation of agricultural operations (as well as migration of natural plant communities) to higher latitudes where plants experience cooler temperatures and longer photoperiods in the summer [5,6,7,8]. Crops utilized for these strategies must be capable of high productivity in environments with varying levels of exposure to cold temperatures. Even in regions where freezing temperatures are uncommon, occasional freezing events must be tolerated. Moreover, of specific importance for productivity is the ability to make adjustments that allow maintenance of high photosynthesis rates under cool temperatures, which is the focus of this review.

Plants that grow as winter annuals germinate in the fall and overwinter before reproducing in the spring. As such, winter annuals must be able to withstand freezing temperatures [9,10,11] as well as maintain high photosynthetic activity under cool temperatures. The catalytic rate of key photosynthetic enzymes is lowered at low temperatures, and the same presumably applies to proteins associated with photosynthate export from leaves (such as SWEET transporters, ATPases, and sugar-loading transporters). To compensate, plant acclimation to low growth temperature involves the development of new leaves with greater maximal photosynthetic capacity as a result of greater levels of photosynthetic protein [12,13,14] and presumably transport proteins. Such a corresponding increase in the number of sugar-export proteins was proposed to take place in winter annuals during acclimation to cool temperature [15]. Plants of the Col-0 genotype of *Arabidopsis thaliana* assessed for their photosynthesis rate (light- and CO_2_-saturated rate of oxygen evolution) under a cool temperature of 12.5 °C exhibited a very low rate of only around 10 µmol O_2_ m^−2^ s^−1^ after being grown under a temperature of 25 °C with moderate PFD and an only slightly higher rate when grown under 25 °C with a high PFD [16]. In contrast, in plants that had developed under (and were fully acclimated to) a cool temperature (leaf temperatures of 12–16 °C) with either moderate or high PFD, much greater (up to four times higher) rates of photosynthesis measured under 12.5 °C were exhibited [16]. This acclimation involved a major infrastructural change at the leaf level, with thicker leaves containing additional layers of chloroplast-rich palisade cells [16,17,18], as is also seen in other herbaceous species that are active during the winter [16,19,20,21,22]. These leaf morphological adjustments are accompanied by larger (sugar-loading) minor leaf veins containing a greater number of sugar-loading cells [16,17,23,24,25,26].

These latter leaf-level anatomical and morphological adjustments of winter annuals in response to cool growth temperatures presumably allow the maximization of the number of photosynthetic and transport proteins per unit of leaf area. In contrast, summer annuals exhibit neither pronounced photosynthetic upregulation nor an increase in palisade layer number or larger minor veins when grown under cool versus warm temperatures [16].

### 1.2. Comparative Ecophysiology and the Study of Phenotypic Plasticity: Historical Perspective and Choice of Study System

**Figure 1:**
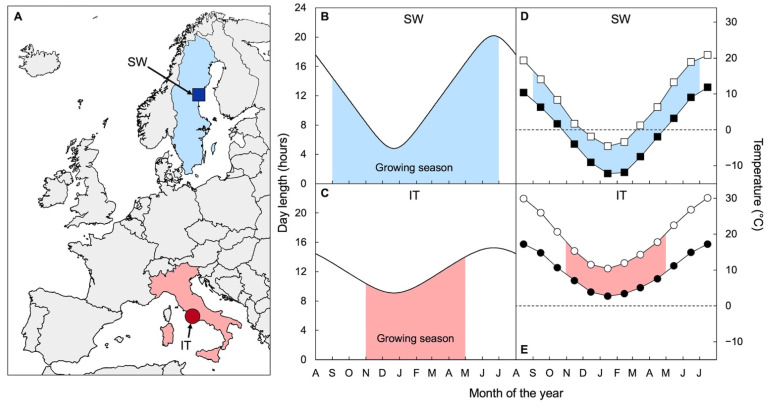
(**A**) Map showing the geographic origins of the SW (blue square; 62°48′ N, 18°12′ E) and IT (red circle; 42°07′ N, 12°29′ E) *A. thaliana* ecotypes (after [37]) as well as (**B**,**C**) the change in day length and (**D**,**E**) monthly minimal (closed symbols) and maximal (open symbols) temperatures across the growing season (light blue or red shading) for (**B**,**D**) SW and (**C**,**E**) IT at their respective sites of origin. Data on growing seasons for the two ecotypes from [41,46].

The use of comparative ecophysiology, i.e., a compare-and-contrast approach to traits of species or populations adapted to contrasting environments, was championed at Stanford’s Department of Plant Biology of the Carnegie Institution of Washington by the group of Jens C. Clausen, David D. Keck, and William M. Hiesey (e.g., [27,28,29]), who built on work by Göte Turesson [30,31,32]. This approach was carried forward by Olle Björkman (e.g., [12,33,34]) and the next group of ecophysiologists at Stanford and others, such as C. Barry Osmond (e.g., [35]), around the world (see [36]). In recent years, this approach was continued by former students of the latter groups and their own mentees, including the authors of this review (see also [36]). As beneficiaries of Olle’s mentorship, we honor his memory as well as his influence and inspiration that loom large in the present work. When we (B.D.-A. and W.W.A.) told Olle that our group would study photosynthetic performance in two *A. thaliana* populations from Sweden (SW) and Italy (IT), he chuckled and predicted that these annual plants would each grow in relatively similar microenvironments and, furthermore, with adjusted phenologies, all of which would minimize the latitude-dependent contrast in overall climatic conditions. If that were the case, it would undoubtedly complicate our effort to identify any physiological differences in photosynthetic performance under common controlled conditions. Fortunately, we had the advantage of Doug Schemske’s and Jon Ågren’s wisdom, as they had picked the specific locations (Figure 1; [37]) and the specific SW (Rodasen-47) and IT (Castelnuovo-12) lines of *A. thaliana* that are the focus of this review. After Ågren and Schemske [37] published evidence of local adaptation from reciprocal transplants, establishing these populations as true “ecotypes” (see [38]), their teams provided valuable and detailed characterizations of these ecotypes under field conditions [39,40,41,42,43,44,45,46], including their growing seasons with the timing of germination, flowering, and seed-set, which turned out to be quite different—just as Olle had predicted.

Overwintering annuals must contend with the prospect of freezing events in many of the environments in which they are found. The SW ecotype faces subfreezing average monthly minimum temperatures in its native habitat for multiple winter months, but IT does not face any subfreezing average monthly minimum temperatures in its native habitat (Figure 1). Perhaps not surprisingly, SW exhibits significantly greater freezing tolerance than IT when grown under common controlled conditions [47,48,49,50,51,52]. The other key element to plant success is the ability to gain enough carbon to support reproduction during periods conducive to productivity [53,54,55]. IT is exposed to average monthly maximal temperatures that remain above 10 °C throughout its life cycle in its native habitat (Figure 1), including the winter months between seedling establishment in early November and seed maturation in late April [46]. On the other hand, SW exhibits an extension of its life cycle, with seedlings establishing much earlier (early September; [46]) and seeds maturing much later (late June; [46]) than IT (Figure 1; see also [41]). As a result, SW and IT experience similar temperatures during corresponding developmental stages (following germination and before reproduction) despite different lifespans. As Olle had predicted, these ecotypes clearly do shrink the differences in environmental conditions they have to contend with for productivity by virtue of their different phenology. These differences were linked to gene loci associated with delayed flowering time in SW [38,41].

The extension of SW’s life cycle into the summer at high latitude also brings with it exposure to much longer days leading up to reproduction (Figure 1) and further exacerbates the latitude-dependent differences in daylength between the ecotypes’ native habitats. This difference represents a greater amplitude of variation in light supply (via photoperiod) in Sweden, with relatively shorter days in winter and even longer days in summer at the site of origin of SW. IT completes its life cycle over a relatively shorter period (fewer months) between fall and spring and is finished by the end of April (Figure 1; [28,29,30]). Agricultural strategies that involve a move to a higher latitude will likewise expose plants not only to seasonally cooler temperatures but also to an increased total light supply with longer days in the summer [5,6,7,8].

Findings related to productivity traits for SW and IT point to a greater degree of phenotypic plasticity in these traits in SW, as the ecotype from the habitat with the greater amplitude in temperature and light over the year/growing season when grown under contrasting light intensities and/or temperatures under controlled conditions [17,24,56,57,58,59,60]. These productivity-related traits chiefly include photosynthetic capacity and associated processes of photosynthate export. However, a different suite of traits exhibited the opposite pattern—greater upregulation of genes that have functions in reactive oxygen species (ROS) detoxification in IT versus SW for comparison of plants grown under low light and warm temperature (LLW) versus high light and cool temperature (HLC) [52]. These conditions consisted of (LLW) 100 µmol photons m^−2^ s^−1^ with leaf temperatures of 25 °C (9-h photoperiod)/20 °C (dark) versus (HLC) 1000 µmol photons m^−2^ s^−1^ and temperatures of 16 °C (9-h photoperiod)/12.5 °C (dark) under common controlled conditions.

The study of phenotypic plasticity (degree of variation in phenotype in response to the environment) in locally adapted populations of other species revealed similarly diverse patterns. It was concluded that the degree of plasticity varies trait by trait among ecotypes [61,62], indicative of the diverse strategies plants employ to survive in specific environmental contexts [63], which may make “local studies … more informative than global ones” [64]. Stearns [65] suggested revising the assessment of traits as either positive or negative by updating the concept of tradeoffs to include a more fluid view of “dynamic linkages.” Similarly, Kleyer and Minden [66] reasoned that the study of single traits does not offer a complete picture and that coordination of traits at the level of the whole plant is necessary. Sack and Buckley [67] also emphasized the need to consider interactions among multiple genes/traits. Reich [68] proposed that integration at the whole-plant level of how traits respond to the environment may create a generalizable response. Combining genomic/transcriptomic information with information on the response of functional traits may further aid in gaining a holistic understanding of plant response [69]. It was proposed that an essential next step in the quest to understand how plants respond to different environments is the identification of underlying themes of how genotypes interact with the environment [70].

**Figure 2:**
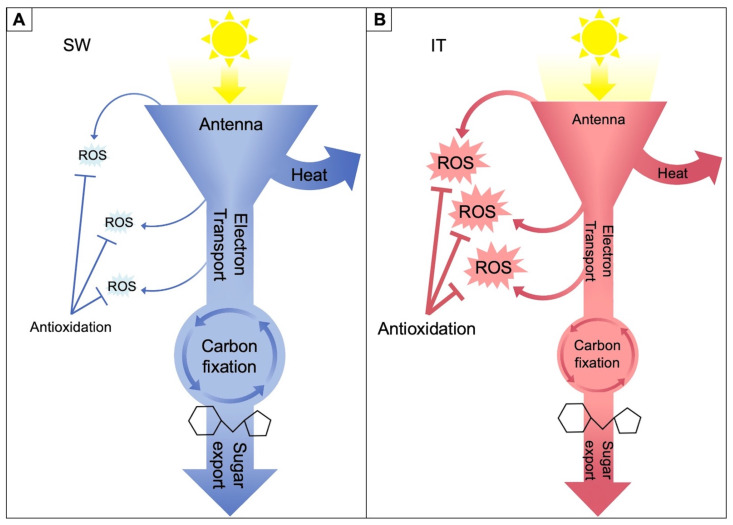
Schematic depiction of relative differences in capacities for light-harvesting (antenna), photoprotective dissipation of excess excitation energy as heat, electron transport, carbon dioxide fixation, sugar export (sucrose symbol), and ROS production and detoxification in leaves of the (**A**) SW and (**B**) IT ecotypes of *A. thaliana*.

In the present review, evidence from multiple studies on the pair of IT and SW ecotypes is summarized in a schematic model (Figure 2) that integrates the responses of suites of traits related to various aspects of photosynthetic productivity and/or protection against oxidative stress in SW versus IT. The schematic depiction in Figure 2 presents these functional features at a glance, positing that SW effectively minimizes excitation pressure and the associated formation of ROS in the chloroplast by pronounced upregulation of the capacity for photosynthetic electron transport, the capacity for preemptive dissipation of excess excitation as heat (thermal energy), and the capacity to export photosynthate from source leaves (Figure 2A). In contrast, it is proposed that IT shifts emphasis, with a somewhat less pronounced capacity for photosynthetic upregulation, pre-emptive dissipation of excess excitation, and exporting photosynthate from source leaves and a proportionally greater emphasis on alternative approaches, including a smaller light-harvesting antenna and pronounced upregulation of multiple antioxidant enzymes that may prevent disruption of cellular redox homeostasis despite formation of more ROS (Figure 2B). Each of these features will be visited in greater detail below.

IT’s different acclimation compared to that of SW may be well suited for IT’s native environment with relatively consistent and mild weather during the plant’s growth cycle. These warm periods may only be interrupted by occasional brief cold periods, followed by rapid resumption of photosynthetic activity when temperatures rise again. Plant productivity in such a scenario may not require the same pronounced up-regulation of maximal photosynthetic capacity as seen in SW. In contrast, the continuously cold conditions in SW’s native habitat may require maximization of the up-regulation of photosynthetic capacity and maintenance of a larger light-harvesting antenna along with a very high capacity for flexible preemptive dissipation of excess excitation as heat from this antenna as needed.

The next section focuses on traits of IT and SW associated with plant productivity, especially the maintenance of photosynthetic activity under cool temperatures in fall and spring. These features are compared in plants of SW and IT that had developed under, and were fully acclimated to, various combinations of temperature and light intensity under controlled growth conditions. The section following that focuses on selected findings from the transcriptomic analysis. SW and IT are characterized as differing in a transcription factor implicated in cold-temperature acclimation [48], as also noted for other *A. thaliana* populations between colder and warmer regions [11,71,72]. The transcription factor is a member of the C-repeat-binding factor (CBF) family of transcription factors (CBF1, CBF2, CBF3; hereafter CBF1–3) that support cold tolerance and high photosynthetic rates in plants grown under cool temperatures and/or high light [53,54]. The *CBF1–3* region was identified as one of the quantitative trait loci (in addition to those associated with flowering time) for fitness in the native habitats of SW and IT [39], which is consistent with the results of other comparisons of *A. thaliana* populations across temperature gradients [11,71].

Subsequent genetic analyses revealed that IT has a mutation in the *CBF2* gene that renders its corresponding CBF2 transcription factor protein nonfunctional [48]. In addition, there are considerably fewer genes in the CBF1–3 regulon in IT versus SW [49]. However, from studies with CBF1–3-deficient lines of IT and SW [49,52], gene regulators other than CBF1–3 transcription factors also play a substantial role in orchestrating the acclimation of both IT and SW to cold temperatures with respect not only to freezing tolerance [49,52] but also photosynthetic acclimation [52]. Likewise, CBF2-deficient lines of SW continued to exhibit greater freezing tolerance than IT [49,51].

## 2. Differential Upregulation of Photosynthesis and Photoprotection, Sugar-Export Infrastructure, and Expression of Relevant Genes

### 2.1. Functional and Anatomical Traits

**Figure 3:**
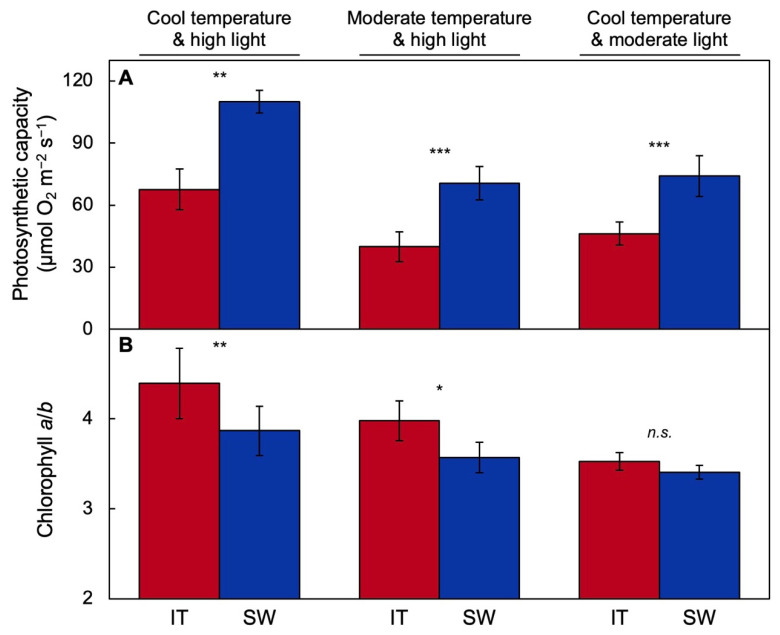
(**A**) Light- and CO_2_-saturated capacity of photosynthesis measured at 25 °C and (**B**) chlorophyll *a*/*b* ratio in IT (red columns) and SW (blue columns) plants grown under a cool temperature and high light (data from [52]), moderate temperature and high light (data from [59]), and cool temperature and moderate light (data from [24,57]). Mean values ± standard deviations (*n* = 3 or 4); statistically significant differences between ecotypes based on Student’s *t*-tests are indicated with asterisks (* = *p* < 0.05; ** = *p* < 0.01; *** = *p* < 0.001); *n.s.* = not significantly different.

As suggested in Figure 2, SW exhibits more pronounced upregulation of photosynthesis than IT (Figure 3A) when grown under a combination of cool temperature and high light [52], moderate temperature and high light [17], or cool temperature and moderate light [58]. SW also maintains a larger light-harvesting antenna than IT under the first two growth conditions, as evidenced by a lower chlorophyll *a*/*b* ratio (Figure 3B).

**Figure 4:**
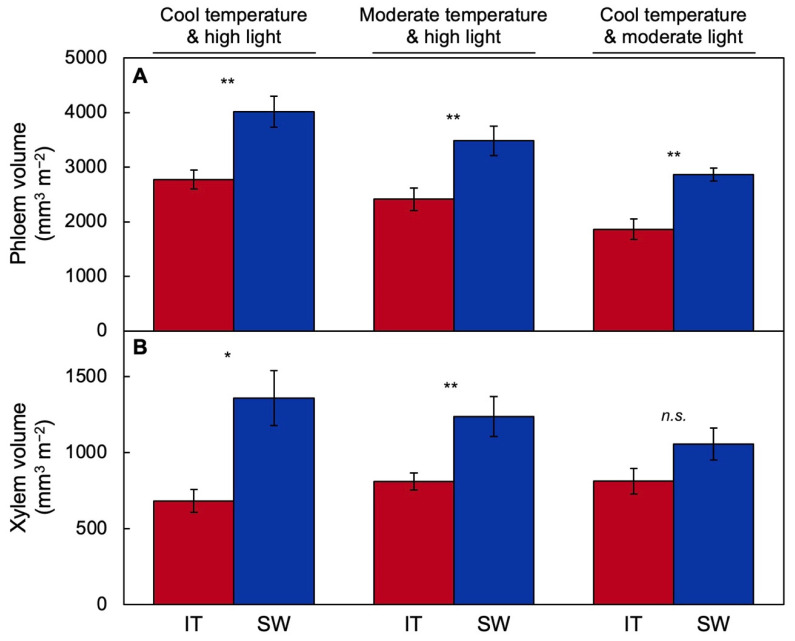
Minor-vein (**A**) phloem and (**B**) xylem volumes on a leaf area basis for IT (red columns) and SW (blue columns) grown under a cool temperature and high light (data from [24]), moderate temperature and high light (data from [59]), and cool temperature and moderate light (recalculated data from [24]). Mean values ± standard deviations (*n* = 3 or 4); statistically significant differences between ecotypes based on Student’s *t*-tests are indicated with asterisks (* = *p* < 0.05; ** = *p* < 0.01); *n.s.* = not significantly different.

Expression of photosynthetic genes is known to respond to sink activity [73], i.e., the combined consumption of carbohydrates by growing and metabolizing tissues and storage of carbohydrates in any storage organs [55]. Maintenance of a high photosynthetic capacity thus requires a high growth rate and/or a high storage capacity. Overwintering annuals limit their above-ground vegetative growth during the winter and presumably store carbohydrates produced in photosynthesis on mild winter days to support a quick resumption of growth in the spring. When grown under cool temperature, SW exhibits more pronounced upregulation than IT of the foliar vascular infrastructure for moving photosynthate out of the leaf (Figure 4A) that match the trends seen for photosynthetic capacity and antenna size (Figure 3). Photosynthate export requires sugar loading and export from the leaf via the phloem (Figure 4A) as well as water transport into the leaf via the xylem (Figure 4B) to replace water lost in transpiration and also move water into foliar phloem in support of the outward-flow of sugar sap (Figure 4B) [24,26,60,74,75]. Similar differences were also reported for plants of SW compared to IT grown under moderate temperatures in high light intensity [17] and cool temperatures in moderate light [58]. The finding of a greater export capacity for photosynthate (sucrose) in SW versus IT is consistent with a greater level of expression of sucrose synthase in plants of SW versus IT grown under HLC [52].

These findings indicate that the pronounced acclimation that allowed maintenance of the photosynthetic capacity and photosynthate export from leaves involved not only greater numbers of photosynthetic and transport proteins but also an expansion of the available leaf infrastructure to accommodate additional chloroplasts and additional vascular cells with sugar-loading transporters.

**Figure 5:**
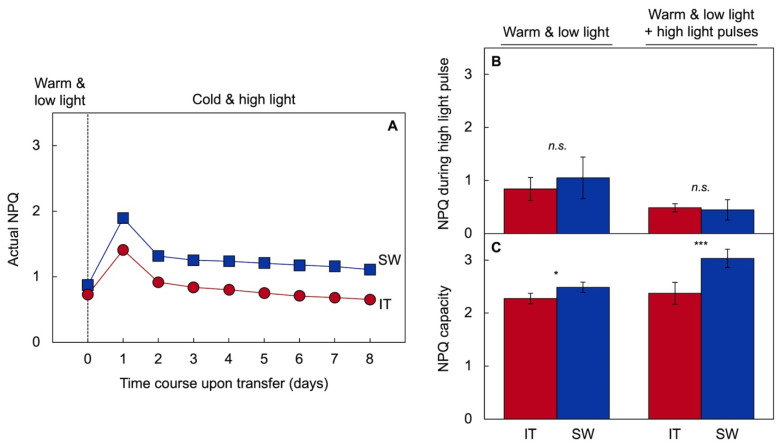
Thermal energy dissipation quantified from non-photochemical quenching (NPQ) of chlorophyll fluorescence in IT (red circles and columns) and SW (blue squares and columns) ecotypes (**A**) upon transfer to cold temperature (4 °C) and high light (800 µmol photons m^−2^ s^−1^) following growth at warm temperatures and low light (re-graphed from [50]), and (**B**) at the end of a 5 min exposure to high light (800 µmol photons m^−2^ s^−1^) as well as (**C**) under measuring conditions of saturating light (over 2000 µmol photons m^−2^ s^−1^) following growth at warm temperature (25 °C) and low light (200 µmol photons m^−2^ s^−1^) with and without 5 min daily high light periods (pulses) (data from [56]). Mean values ± standard deviations (*n* = 4); statistically significant differences between ecotypes based on Student’s *t*-tests are indicated with asterisks (* = *p* < 0.05; *** = *p* < 0.001); *n.s.* = not significantly different.

Figure 5 depicts non-photochemical quenching (NPQ) of chlorophyll fluorescence as a measure of pre-emptive dissipation of excess excitation as heat [76] in SW and IT. Figure 5A shows that the level of NPQ was greater in SW versus IT upon transfer of warm-grown plants to cold temperatures (4 °C) under high light levels of 800 µmol photons m^−2^ s^−1^ [50]. A clear increase in the capacity for thermal energy dissipation is induced by acclimation to even moderate levels of excess light in the absence of cool temperatures (Figure 5C). Moreover, Figure 5 panels B,C indicate that such a greater capacity for thermal energy dissipation may be most evident under very high light levels at moderate temperature. Figure 5 panels B,C show NPQ in plants of both ecotypes grown under rather low light levels of 200 µmol photons m^−2^ s^−1^ either in the absence or presence of five daily 5 min exposures to 800 µmol photons m^−2^ s^−1^ (high light pulses; [56]). Actual NPQ levels were similarly low in SW and IT plants (Figure 5B) when measured during 5 min exposure to 800 µmol photons m^−2^ s^−1^. In contrast, maximal NPQ capacity determined under experimental exposure to a much higher measuring intensity of over 2000 µmol photons m^−2^ s^−1^ revealed a substantially greater maximal capacity for thermal energy dissipation in SW compared to IT in plants grown with daily 5 min high light pulses [56]. Again, this indicates that a higher NPQ capacity in SW is inducible by acclimation to environmental stress that involves excess excitation (due to transfer to cold conditions or regular exposure to even modest levels of excess light during plant development). It should be noted that excess light results from an imbalance in light level (i.e., input of excitation) versus temperature (as affecting utilization of excitation), resulting in excess excitation pressure under very high or fluctuating light even in the presence of moderate temperature (or modestly cool temperatures above the chilling range) or, conversely, under colder temperatures in the presence of low light levels.

**Figure 6:**
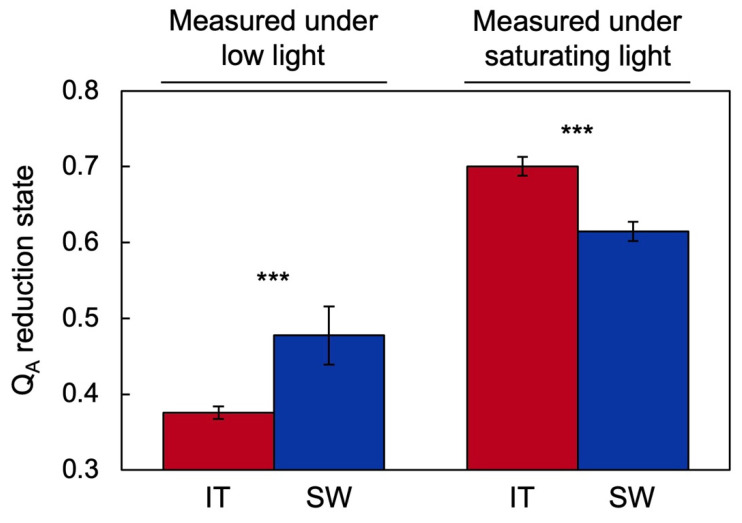
Excitation pressure as assessed from the reduction state of the primary electron acceptor Q_A_ of photosystem II (via chlorophyll fluorescence [77]) measured under either low light (50 µmol photons m^−2^ s^−1^) or saturating light (2000 µmol photons m^−2^ s^−1^) in IT (red columns) and SW (blue columns) grown under cool temperature and high light. Mean values ± standard deviations (*n* = 3); statistically significant differences between ecotypes based on Student’s *t*-tests are indicated with asterisks (*** = *p* < 0.001). Data from [52].

Figure 6 depicts the level of excitation pressure in the chloroplast as assessed from the reduction state of the primary electron acceptor Q_A_ of photosystem II via chlorophyll fluorescence [52]. Under a low measuring light intensity, excitation pressure was lower in IT versus SW plants grown under high light and cool temperatures, which is consistent with the lower light-harvesting capacity (smaller antenna with higher chlorophyll *a*/*b* ratio) in IT grown under these conditions (Figure 3). In contrast, under a high measuring light intensity, the situation was reversed with a lower excitation pressure in SW, which is consistent with the higher capacity for both the thermal dissipation and photochemical utilization of excitation energy in HLC-grown SW. This finding further supports the model shown in Figure 2 and suggests that higher levels of ROS may be formed in IT versus SW under these conditions.

### 2.2. Differential Gene Expression

**Figure 7:**
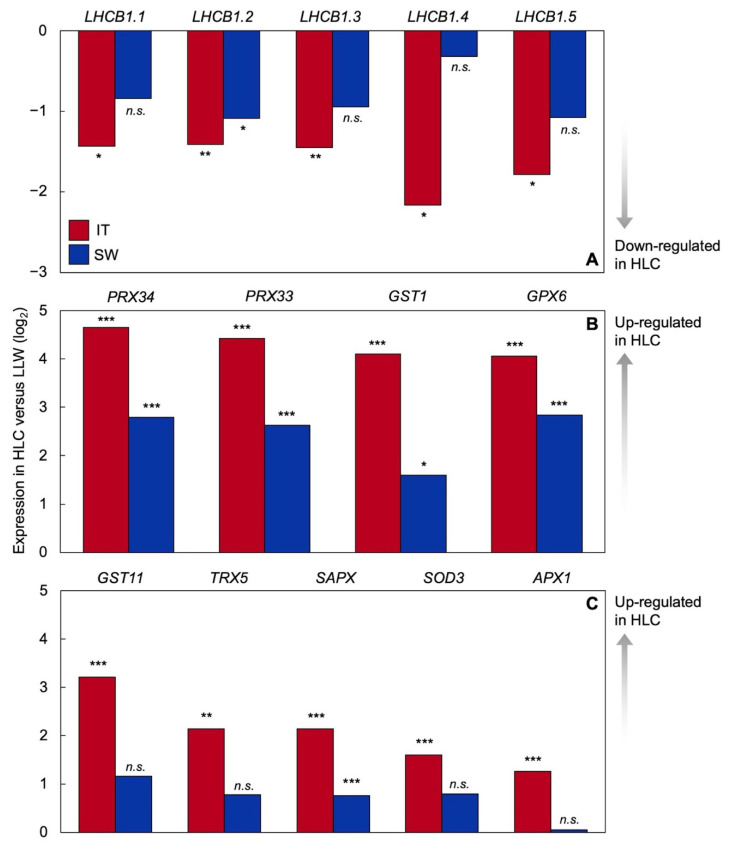
Gene expression patterns of either up- or downregulation in plants of IT and SW grown under high light and cold temperature (HLC) versus low light and warm temperature (LLW) for (**A**) several proteins of the light-harvesting chlorophyll (*a* + *b*)-binding (LHCB) family, including *LHCB1.1* (AT1G29920); *LHCB1.2* (AT1G29910); *LHCB1.3* (AT1G29930); *LHCB1.4* (AT2G34430), and *LHCB1.5* (AT2G34420) as well as (**B**,**C**), selected antioxidant enzymes, including class III peroxidases *PRX33* (AT3G49110) and *PRX34* (AT3G49120), glutathione S-transferases *GST1* (AT1G02930) and *GST11* (AT1G02920), glutathione peroxidase *GPX6* (AT4G11600), cytosolic thioredoxin *TRX5* (AT1G45145), stromal ascorbate peroxidase *SAPX* (AT4G08390) and ascorbate peroxidase *APX1* (ATG07890), and iron superoxide dismutase *SOD3* (AT5G23310). Significant up- or down-regulation between growth conditions are indicated with asterisks (* = *p* < 0.05; ** = *p* < 0.01; *** = *p* < 0.001); *n.s.* = not significantly different. Data from [52].

Figure 7 shows differential gene regulation patterns for IT and SW as the degree of down- or up-regulation in plants grown under HLC compared to LLW [52]. Figure 7A shows significant downregulation of several light-harvesting chlorophyll (*a* + *b*)-binding (*LHCB)* genes in HLC-grown IT but no significant downregulation in SW in four of the five *LHCB* genes. This finding is consistent with the chlorophyll *a*/*b* ratios shown in Figure 4 that indicate a smaller antenna size in HLC-grown IT versus SW as well as with the lower reduction state of Q_A_ in IT, indicative of lesser excitation pressure under high light exposure (Figure 6). Moreover, panels B and C of Figure 7 show consistently pronounced upregulation in HLC versus LLW in IT for a host of genes encoding antioxidant enzymes that serve in ROS detoxification. In contrast, in SW, some of these genes either did not exhibit significant upregulation or were upregulated to a lesser extent in HLC-grown versus LLW-grown plants. A more pronounced upregulation of antioxidant enzymes in HLC-grown versus LLW-grown IT is consistent with the model in Figure 2, suggesting higher levels of ROS production resulting from a higher excitation pressure under high light exposure in IT versus SW (Figure 6) that is apparently addressed by pronounced ROS detoxification in IT. Several additional genes with roles in maintaining redox homeostasis are highlighted by Baker et al. [52].

These findings support a proportionally greater emphasis on the mitigation of oxidative stress in IT as the ecotype that exhibits a lesser combined capacity for utilization (in electron transport) and pre-emptive dissipation (as heat) of excitation energy than SW (see also Figure 2). This finding, furthermore, suggests that antenna size in IT is not sufficiently downsized to keep ROS formation from rising more in IT than in SW. The concomitant upregulation of ROS detoxification may offer the benefit of appreciable light absorption and utilization when possible (under less cool temperatures) while maintaining redox homeostasis by virtue of ROS detoxification when needed (during a cold spell). One can think of this difference in the strategies of SW and IT as SW opening a pre-emptive overflow valve (thermal dissipation) in the bathtub, while IT waits to see if the tub overflows (with ROS) and then simply mops the floor with a stack of absorbent towels (ROS-detoxifying antioxidant enzymes). It is tempting to speculate that this difference is associated with the difference in frequency of how often excitation energy tends to become excessive (i.e., how frequently the bathtub threatens to overflow) in the ecotypes’ respective native habitats.

## 3. Growth Patterns as Affected by Growth Environment and Genotype

Winter annuals slow their growth under short days and cool temperatures, which is thought to be protective in limiting frost damage to overwintering shoots [78]. CBFs cause stunting of growth as evidenced, e.g., by the growth inhibition seen in plants that overexpress CBF (e.g., [49]).

SW and IT both exhibited slow growth under cool temperatures (leaf temperatures of 12–16 °C), with a significant, albeit rather small, difference between IT and SW (slightly smaller rosette in SW; [52]). Likewise, plants grown under an even colder temperature of 4 °C showed a similar difference between IT and SW both in rosette area and dry biomass [49], again with SW exhibiting slower growth. The slowing of growth under cool temperature was significantly released in the CBF1–3-deficient mutant of IT but to a lesser (albeit significant) extent in the CBF1–3-deficient mutant of SW [49,52]. Many of the phenotypic acclimatory responses to cold temperature and/or high light were still seen in CBF1–3-deficient mutants, again indicating that other regulators can fulfill the role of the CBF1–3 transcription factors [49,52]. More research is needed to assess the role of CBF2 and other regulators in the ability of IT to maintain a similar growth suppression as seen in SW under cool growth temperatures and, unlike SW, release growth suppression under hot growth temperatures.

In stark contrast, the difference in growth between wildtype IT and SW was dramatic under either low versus high growth light intensity (100 vs. 1000 µmol photons m^−2^ s^−1^) under moderate temperature [17] or under hot temperature (leaf temperature of 36 °C during the light period and 25 °C during the dark period, respectively) versus cool temperature (leaf temperatures of 14 °C during the light period and 12.5 °C during the dark period, respectively) under moderate light during plant growth [58]. In either case (low growth light or hot growth temperature), IT exhibited dramatically larger rosettes that were twice the size as those of SW. Again, no such differences were seen in high light or cool temperatures [17,58].

The take-home message from these latter findings is that both IT and SW exhibited the expected, and presumably protective, stunting of growth under cool growth temperatures, but that only IT (and not SW) released growth inhibition under hot temperatures. These differences can be viewed in the context of the different environmental conditions during the growing season of the two ecotypes in their respective native habitats. Growth suppression during the short winter season in Italy should protect IT against damage during any freezing episodes that are rare events in IT’s native habitat [37]. Resumption of rapid growth in early spring should aid in the completion of IT’s life cycle before the summer. In fact, the stimulation of IT’s growth by warm temperature could be viewed as akin to the response of desert ephemerals that accelerate, rather than slow, growth (toward rapid completion of their life cycle) at the first signs of rising temperatures in late spring [79]. Conversely, even mid-summer conditions in Sweden do not include any particularly warm temperatures, and an ability to speed up growth and hasten life-cycle completion in response to rising temperatures would appear to be of no benefit to SW.

## 4. Summary and Conclusions in the Context of Climate Resilience and Dynamic Linkages

As summarized in the schematic model shown in Figure 2, SW exhibited particularly strong upregulation of features that support the maintenance of photosynthetic productivity under cool temperatures, thereby effectively limiting increases in excitation pressure and the associated formation of excess ROS. These features of SW included a greater upregulation of the capacity to utilize excitation energy via photosynthetic electron transport, of preemptive dissipation of excess excitation as heat, and of the foliar vascular infrastructure for photosynthate export. In addition, SW largely maintained its antenna size when grown under HLC. Conversely, IT exhibited significant downregulation of its light-harvesting antenna size and a proportionally greater upregulation of a broad range of enzymatic antioxidation processes that counter oxidative stress under HLC compared to LLW.

Concerning growth rates, SW and IT exhibited a similar slowing of growth under cool temperature, but not under warm/hot growth temperature, where IT but not SW released the growth arrest. Each ecotype thus demonstrates specific productivity-related features of life cycle progression in their natural habitat and photosynthetic performance and growth under common environmental conditions. The adaptations in leaf form and function seen in SW presumably offer (i) particularly strong support for the maintenance of photosynthetic activity and export of photosynthate from leaves under continuously cool temperature, which minimizes excess-ROS formation, and (ii) protection against frost damage of above-ground tissue by capping rosette growth. Conversely, the adaptations of IT from a native habitat with extended periods of warmer temperatures during winter involve releasing constraints on growth under warm temperature and/or low light while still maintaining growth caps under cool temperature. IT’s response to growth under HLC involves a combination of downsizing of light-harvesting antenna size (i.e., less capture of light for utilization in photosynthesis) and proportionally more pronounced upregulation of ROS-detoxifying antioxidation processes.

This summary aims to integrate traits/genes and place them into the context of plant performance in specific environments. In particular, this analysis suggests that the lesser degree of freezing tolerance and the lesser upregulation of photosynthetic capacity in IT should not be viewed as inferior to that of SW. It rather appears that the different strategies of IT could be seen as highly suitable for an environment with a low probability of freezing events and substantial periods of time for photosynthetic productivity under temperatures above the chilling range. Particularly strong upregulation of an array of ROS-detoxifying antioxidant enzymes under exposure to cool temperature can be seen as an effective and efficient way to avert oxidative damage if/when needed while tolerating a dip in photosynthetic productivity during a brief cold spell in favor of the resumption of photosynthesis in the subsequent warmer period. The acceleration of growth during rising temperatures may have benefits in environments that experience summer heat. Overall, the difference between IT and SW could be characterized metaphorically as SW pushing forward at full speed with productivity under cool temperatures and IT staying safe from harm while taking a bit of a break from productivity when temperatures are cool.

In conclusion, both ecotypes undergo acclimation to growth under cool temperature that meets the criterion for full acclimation as a transition from an experience of “stress” (with a temporary loss of redox balance resulting in oxidative stress) to a new steady-state in which little to no endogenous stress is experienced (with restored redox balance), and where excessive ROS levels are avoided by a combination of pre-emptive mechanisms that counteract ROS formation (less light absorption and/or boosted utilization/thermal dissipation of excitation) and removal of ROS once formed (detoxification by antioxidant enzymes). The difference between IT and SW lies in the relative proportion to which pre-emptive and detoxification mechanisms are employed to reach acclimation. Experimental conditions for the testing of additional genotypes should involve not only the sudden transition of unacclimated leaves to stress conditions but also time periods long enough to allow for the development of new leaves under the new conditions.

Preliminary lessons from this insight into productivity-related traits of IT and SW for agriculture include:

1. The movement of agriculture to a cooler time of the year or a higher latitude, as an increasingly discussed agricultural approach [5,6,7,8], should be accompanied by crop development for each specific scenario. The design of climate-resilient crops should take into consideration the specific target latitude and associated degree of environmental variability.
2. The development of improved crops for cultivation at high latitudes (with continuously low temperatures) should probably follow the acclimation pattern of SW, whereas improved crops for mid-latitudes (where cold spells are only intermittent) should probably follow the acclimation pattern of IT.
3. Whereas this review focused on the comparison of two ecotypes of a winter annual, a comparison of either of these with summer annuals may be informative. It should be assessed whether IT, but not SW, combines traits of a winter annual (upregulation of freezing tolerance and photosynthetic and photosynthate-export capacity under cool growth temperatures) with those of a summer annual. IT’s higher growth rate in warm temperatures supports such a notion.
4. More generally, environments with continuous exposure to cool temperatures that dramatically lower productivity in unacclimated plants may be best met with acclimatory adjustments of plant form and function supporting the maintenance of photosynthetic productivity and sugar export from source leaves, thereby pre-emptively minimizing shifts in cellular redox homeostasis. In contrast, environments with extended periods of warmer temperatures and the possibility of only brief interspersed cold spells may be adequately met with a relatively greater emphasis on upregulation of genes with roles in the maintenance of cellular redox homeostasis during intermittent periods of stress. Pronounced growth acceleration when temperatures rise in the spring may play a role in evasion of summer heat.
5. Moreover, the trajectory from initial plant exposure to cold temperatures all the way to full acclimation (with leaves newly grown and fully developed under these conditions) likely involves a more pronounced transient upregulation of the antioxidation processes even in genotypes that eventually fully re-establish photosynthetic productivity. Leaves of winter annuals may thus rely to a relatively lesser degree on management rather than avoidance of oxidative stress in fully acclimated new leaves that have developed under cool growth temperatures.
